# Second-Trimester Fibrinogen-to-Albumin Ratio and Platelet Activation Markers Compared with the HALP Score for Predicting Preeclampsia

**DOI:** 10.3390/diagnostics16040613

**Published:** 2026-02-19

**Authors:** Cagla Bahar Bulbul, Betul Yakistiran

**Affiliations:** 1 Medical Park Izmir Hospital, Department of Obstetrics and Gynecology, Izmir 35230, Turkey; 2 Balikesir Ataturk City and Research Hospital, Department of Obstetrics and Gynecology, Balikesir 10100, Turkey

**Keywords:** preeclampsia, fibrinogen-to-albumin ratio (FAR), platelet activation markers, hemoglobin–albumin–lymphocyte–platelet (HALP) score

## Abstract

**Objective:** This study aimed to evaluate the predictive value of second-trimester hemoglobin levels, the hemoglobin–albumin–lymphocyte–platelet (HALP) index, the fibrinogen-to-albumin ratio (FAR), and selected coagulation and platelet activation markers for the development of preeclampsia. **Methods:** This retrospective cohort study included 262 pregnant women, comprising 131 women who developed preeclampsia and 131 normotensive controls, followed at a tertiary referral center between 2022 and 2023. Maternal demographic, clinical, and laboratory data were obtained from routine second-trimester (14–28 weeks) antenatal assessments. HALP and FAR were calculated using standardized formulas. Group comparisons were performed using appropriate parametric or nonparametric tests. Discriminative performance was assessed using receiver operating characteristic (ROC) curve analysis with bootstrap resampling. Univariate and multivariable logistic regression models were constructed to evaluate independent associations, and combined biomarker models were compared using DeLong’s test. **Results:** Women with preeclampsia demonstrated significantly lower hemoglobin, hematocrit, platelet count, and albumin levels, alongside higher fibrinogen, D-dimer, LDH, CRP, and platelet activation indices (MPV, PDW, and P-LCR) (all *p* < 0.05). Both HALP and FAR were significantly higher in the preeclampsia group; however, FAR exhibited superior discriminatory ability (AUC 0.682; 95% CI 0.618–0.751) compared with HALP (AUC 0.619; 95% CI 0.551–0.680). In multivariable analysis, FAR remained a strong independent predictor of preeclampsia (adjusted OR 1.263; 95% CI 1.167–1.368), whereas HALP showed a weaker association. Among combined models, FAR plus platelet distribution width (PDW) provided the highest discrimination (AUC 0.820), significantly outperforming FAR alone (DeLong *p* = 0.030). **Conclusion:** While the FAR + PDW model demonstrated improved discriminatory performance, these findings should be interpreted as preliminary. Prospective multicenter studies and external validation are necessary before such biomarkers can be considered for routine clinical use.

## 1. Introduction

Preeclampsia remains a leading contributor to maternal and perinatal morbidity worldwide, affecting approximately 4–6% of pregnancies and increasing the risks of preterm birth, fetal growth restriction, and long-term cardiovascular complications in affected women [1,2]. Preeclampsia is increasingly recognized as a complex multisystem disorder rather than a condition defined solely by hypertension and proteinuria [3].

Preeclampsia is strongly associated with an imbalance between pro-angiogenic and anti-angiogenic factors, reflecting early placental dysfunction. Excess placental release of soluble fms-like tyrosine kinase-1 (sFlt-1) antagonizes vascular endothelial growth factor (VEGF) and placental growth factor (PlGF), leading to impaired angiogenesis, endothelial injury, and widespread maternal vascular dysregulation [4]. This angiogenic disequilibrium contributes to reduced nitric oxide bioavailability, oxidative stress, and placental hypoxia, ultimately triggering systemic inflammatory and coagulation pathways. Although angiogenic biomarkers—particularly the sFlt-1/PlGF ratio—have demonstrated strong predictive performance, their routine clinical use may be constrained by cost, assay availability, and the need for repeated measurements. Placental ischemia further amplifies these processes, contributing to the multisystem clinical manifestations of preeclampsia [5]. Impaired extravillous trophoblast invasion, together with inadequate spiral artery remodeling, represents a fundamental early event in the pathogenesis of abnormal placentation in preeclampsia [6,7]. Failure of these physiological transformations results in high-resistance uteroplacental circulation, promoting chronic placental hypoperfusion and ischemia. This hypoxic microenvironment further exacerbates anti-angiogenic signaling and endothelial dysfunction, reinforcing the systemic maternal inflammatory and procoagulant response characteristic of the disorder. Importantly, these biomarkers predominantly reflect upstream placental dysfunction; however, markers capturing the downstream maternal inflammatory and coagulation responses may provide complementary and clinically actionable insight. Consequently, the overall predictive performance of currently available markers remains suboptimal, underscoring the need for integrated biomarker approaches.

Anemia, lymphocyte dysfunction, platelet activation, hypoalbuminemia, and hyperfibrinogenemia—individually and collectively—have all been implicated in impaired placental perfusion and exaggerated maternal inflammatory responses characteristic of preeclampsia [8,9]. Coagulation abnormalities—including shortened clotting times, increased fibrinogen levels, and subtle platelet consumption—represent another hallmark of hypertensive disorders of pregnancy [10]. Dysregulated coagulation not only contributes to endothelial microangiopathy but may also interact with inflammatory and nutritional pathways, further amplifying disease progression. Given that these laboratory parameters are routinely assessed during antenatal care, their potential use in risk prediction offers notable clinical appeal.

The HALP score (hemoglobin × albumin × lymphocyte/platelet) represents one such multidimensional index, integrating hematologic status, inflammatory tone, and nutritional adequacy. Although originally developed in oncologic prognostication, recent investigations have examined its relevance in obstetrics [11,12,13,14]. Findings, however, remain inconsistent; both low and high HALP values have been observed, likely reflecting differences in disease severity, timing of measurement, or population characteristics in obstetrics [15,16,17,18]. Since predisposition to coagulation is involved in the pathophysiology of preeclampsia, the evaluation of HALP dynamics, especially in the second trimester when systemic inflammation intensifies, constitutes the primary rationale of the present study.

Similarly, the fibrinogen-to-albumin ratio (FAR) is an emerging biomarker reflecting hypercoagulability and inflammation. Elevated FAR has been associated with preterm birth, systemic inflammation, and endothelial injury, yet evidence regarding its relationship with preeclampsia remains limited and inconsistent [17]. Since hyperfibrinogenemia and hypoalbuminemia are characteristic biochemical alterations in preeclampsia, FAR may provide an integrated measure of disease predisposition.

Despite the growing interest in these composite indices, there is a paucity of studies specifically evaluating second-trimester hemoglobin, HALP score, FAR, and coagulation markers as predictors of subsequent preeclampsia. Thus, there is an unmet need for research that systematically investigates whether accessible, noninvasive hematologic and inflammatory markers measured later in gestation can help identify women at elevated risk of developing preeclampsia. While first-trimester screening focuses primarily on placentation-related biomarkers, and third-trimester assessments often coincide with established disease, the second trimester offers a unique window to detect emerging biochemical signatures of endothelial dysfunction, inflammation, and hypercoagulability. Physiological hemodilution stabilizes during this period, platelet turnover accelerates, and inflammatory pathways become increasingly active, rendering hematologic and biochemical markers more reflective of underlying pathology rather than gestational adaptation alone [19]. Despite this biological relevance, second-trimester biomarkers remain underexplored, particularly composite indices derived from routinely available laboratory parameters.

Considering the underlying causes of preeclampsia, a comparative assessment of the HALP score and the FAR index may clarify which composite marker better aligns with the biological mechanisms underlying gestational hypertension disorders. Identifying reliable and easily obtainable biomarkers may enhance early risk stratification and support more individualized antenatal surveillance strategies. We aimed to investigate whether second-trimester hemoglobin levels, HALP score, FAR ratio, and key coagulation-related biomarkers are associated with the development of preeclampsia. By integrating indices that reflect inflammation and coagulation, this study seeks to improve risk stratification and support earlier identification of pregnancies at increased risk.

## 2. Materials and Methods

### 2.1. Study Design and Participants

This retrospective cohort study was conducted at the Department of Obstetrics and Gynecology, Balıkesir Atatürk City Hospital, Türkiye, and included pregnant women admitted for routine antenatal care between 1 January 2022 and 31 December 2023. Ethics approval was obtained from the Balıkesir Atatürk City and Research Hospital Ethics Committee prior to the study, and it conforms to the provisions of the Declaration of Helsinki (Approval No.: 2024/01/4; date of approval: 25 January 2024). Patient consent was waived due to the retrospective design of the study.

Women aged ≥18 years with a singleton pregnancy, who later developed preeclampsia, were eligible for inclusion. Preeclampsia diagnosis was established according to the 2020 American College of Obstetricians and Gynecologists (ACOG) criteria [1]. Exclusion criteria included multiple pregnancies, chronic hypertension, pregestational diabetes mellitus, pre-existing renal or cardiovascular disease, autoimmune disorders (including systemic lupus erythematosus and antiphospholipid syndrome), chronic inflammatory conditions, multiple gestation, conception via assisted reproductive technologies, fetal structural or chromosomal anomalies, and the use of systemic corticosteroids or anti-inflammatory medications during pregnancy. Because laboratory analyses were evaluated retrospectively, clinicians were not aware of the study hypotheses, minimizing potential management bias.

The control group consisted of normotensive pregnant women without proteinuria or systemic disease. Controls were randomly selected from eligible normotensive pregnancies during the same study period to obtain an equal sample size and minimize selection bias. Complete-case analysis was performed; missing data were <5%, and therefore no imputation was required. In total, 131 women with preeclampsia and 131 controls were included. Because all eligible cases were included, a formal a priori power calculation was not feasible. However, post hoc detectable effect analysis indicated that the sample size of 262 participants provides >80% power to detect medium effect sizes (Cohen’s d ≈ 0.35) in continuous biomarkers and AUC differences ≥ 0.07. Bootstrap-based confidence interval width remained stable across resamples, supporting adequate precision of AUC estimates.

### 2.2. Data Collection and Variables

Demographic, clinical, obstetric, and neonatal variables were extracted from electronic medical records by two independent researchers. Recorded parameters included maternal age, gravidity, parity, BMI, smoking status, alcohol use, previous obstetric history, gestational age at delivery, and neonatal outcomes.

Laboratory parameters were obtained from routine antenatal blood tests performed during the second trimester (gestational weeks 14–28). Extracted laboratory variables included hemoglobin, hematocrit, albumin, lymphocyte count, platelet count, fibrinogen, D-dimer, ALT, AST, creatinine, LDH, CRP, and platelet indices (MPV, PDW, and P-LCR). All analyses were performed using the same automated hematology and biochemistry analyzers throughout the study period to ensure consistency. The hemoglobin–albumin–lymphocyte–platelet (HALP) index was calculated using the following formula: HALP = hemoglobin (g/L) × albumin (g/L) × lymphocytes (/L)/platelets (/L) [20]. The fibrinogen-to-albumin ratio (FAR) was calculated as FAR = fibrinogen (g/L)/albumin (g/dL).

The second-trimester time frame for laboratory assessment was deliberately selected to minimize the confounding effects of early gestational physiological variability and late-stage disease-related interventions. During mid-gestation, plasma volume expansion and hematologic adaptations reach a relatively stable plateau, allowing biomarker alterations to more accurately reflect pathological processes rather than normal pregnancy-related changes. To reduce measurement bias, all laboratory analyses were performed using the same automated hematology and biochemistry platforms throughout the study period, and institutional reference ranges remained unchanged. This standardized approach enhances internal validity and supports the comparability of biomarker measurements across the study cohort.

### 2.3. Statistical Analysis

All statistical analyses were performed using MedCalc Statistical v.23.1.5 Software. Normality was assessed using the Shapiro–Wilk test, and variance homogeneity was assessed with Levene’s test. Normally distributed variables were compared using Welch’s *t*-test. Non-normally distributed variables were analyzed using the Mann–Whitney U test. Categorical variables were compared using the chi-square or Fisher’s exact test.

Receiver operating characteristic (ROC) curves were generated to evaluate the discriminative ability of HALP, FAR, and related biomarkers. Area under the curve (AUC) values and 95% confidence intervals were calculated using 200 bootstrap resamples. Optimal cut-off values were determined using Youden’s index. Outliers were evaluated using boxplots and z-scores, and no biologically implausible values were identified. Linearity of continuous predictors with the logit was verified using the Box–Tidwell test with Bonferroni adjustment. Cut-off values derived from combined logistic regression models represent predicted probability thresholds rather than raw biomarker concentrations.

Univariate logistic regression was performed for each biomarker, followed by a multivariable model including HALP and FAR to prevent over-adjustment and isolate biomarker-specific effects. Model calibration was assessed using the Hosmer–Lemeshow goodness-of-fit test, and multicollinearity was evaluated using variance inflation factors. All combined biomarker models were additionally assessed and demonstrated acceptable collinearity (VIF < 2). Although maternal characteristics such as BMI, parity, and smoking status were recorded and compared between groups, they were not incorporated into the primary multivariable models. This analytical strategy was chosen to avoid over-adjustment and to focus on the intrinsic predictive performance of the hematologic biomarkers under investigation. Such an approach allows clearer interpretation of biomarker-specific associations independent of established epidemiologic risk factors.

Correlation analyses were conducted using Pearson coefficients. Boxplots, ROC curves, forest plots, and radar charts were generated for visualization. Group differences shown in boxplots were evaluated using the Mann–Whitney U test. To evaluate whether combinations of biomarkers improved discrimination, multivariable logistic regression models were constructed using FAR together with LDH, PDW, or MPV. Predicted probabilities from each model were used to generate ROC curves, and pairwise comparisons were performed using DeLong’s test.

Univariate logistic regression identified biomarkers associated with preeclampsia. Variables with *p* < 0.10 and biological plausibility were included in multivariate models. Multicollinearity was assessed using variance inflation factors (VIFs), with all predictors demonstrating acceptable values (VIF <2). Combined biomarker models were additionally evaluated and showed VIF <2, indicating no concerning collinearity. Model calibration was assessed using the Hosmer–Lemeshow goodness-of-fit test. Statistical significance was set at *p* < 0.05.

## 3. Results

A total of 262 pregnant women were included in the analysis, comprising 131 women with preeclampsia and 131 normotensive controls. Baseline maternal characteristics are presented in Table 1. Maternal age and BMI did not differ between the groups (both *p* > 0.05). However, women with preeclampsia had significantly lower gravidity and parity values compared with controls (both *p* < 0.05). As expected, systolic and diastolic blood pressures were markedly higher in the preeclampsia group (*p* < 0.05 for both). Gestational age at delivery was slightly lower among cases (*p* < 0.05). Although the between-group difference was clinically modest, the distribution of gestational age was significantly narrower in the preeclampsia group, reflecting reduced variance rather than a clinically meaningful difference in mean gestational age. Cesarean delivery was significantly more common in the preeclampsia group, while the modest difference in neonatal birthweight between groups was not statistically significant (*p* = 0.1667).

### 3.1. Laboratory Findings

Laboratory comparisons between groups are summarized in Table 2. Among hematologic parameters, hemoglobin, hematocrit, platelet count, and platelet indices differed significantly, whereas lymphocytes were comparable between groups. Hemoglobin and hematocrit levels were significantly lower in preeclampsia (both *p* < 0.05). Platelet indices demonstrated marked activation (MPV, PDW, P-LCR all *p* < 0.05), while platelet counts were reduced. Lymphocyte counts, however, were comparable between groups (*p* = 0.4189). Fibrinogen, LDH, and D-dimer values were substantially higher in the study group (all *p* < 0.05). Albumin concentrations were significantly lower in women with preeclampsia (*p* < 0.05). C-reactive protein (CRP) levels were also significantly higher in the preeclampsia group compared with controls, consistent with an enhanced systemic inflammatory response (*p* < 0.05).

The HALP score was higher in the preeclampsia group (47.23 ± 25.56 vs. 37.73 ± 16.51; *p* < 0.05), although with modest discriminatory ability. In contrast, FAR demonstrated a pronounced elevation (12.44 ± 4.43 vs. 9.63 ± 3.27; *p* < 0.05). Serum creatinine levels were significantly higher among preeclamptic women (0.57 ± 0.12 vs. 0.50 ± 0.08 mg/dL; *p* < 0.05).

### 3.2. Correlation Analysis

**Figure 1:**
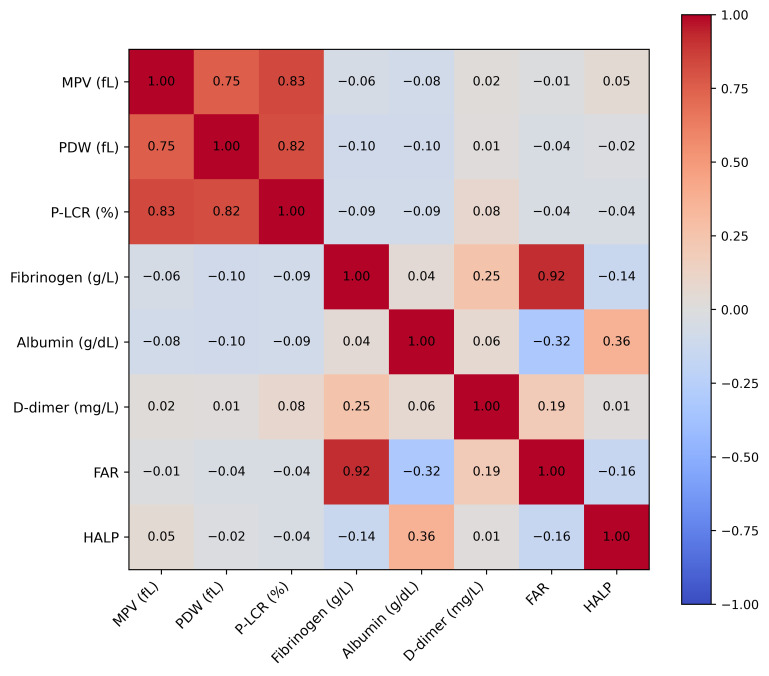
Correlation heatmap illustrating the relationships among platelet indices (MPV, PDW, and P-LCR), fibrinogen, albumin, D-dimer, the fibrinogen-to-albumin ratio (FAR), and the HALP index. Strong correlations were observed among platelet indices and between fibrinogen and FAR. D-dimer showed weak-to-moderate correlations with fibrinogen and FAR, reflecting activation of coagulation pathways. HALP demonstrated minimal correlation with other biomarkers, consistent with its limited predictive performance. Values represent Pearson correlation coefficients.

A comprehensive correlation matrix is shown in Figure 1. Platelet indices were strongly intercorrelated (MPV–PDW r ≈ 0.72; PDW–P-LCR r ≈ 0.65). Fibrinogen correlated strongly with FAR (r ≈ 0.70) and inversely with albumin (r ≈ –0.61). D-dimer demonstrated weak-to-moderate correlations with fibrinogen and FAR (r ≈ 0.30–0.40). HALP showed only weak correlations with all biomarkers (|r| < 0.25).

**Figure 2:**
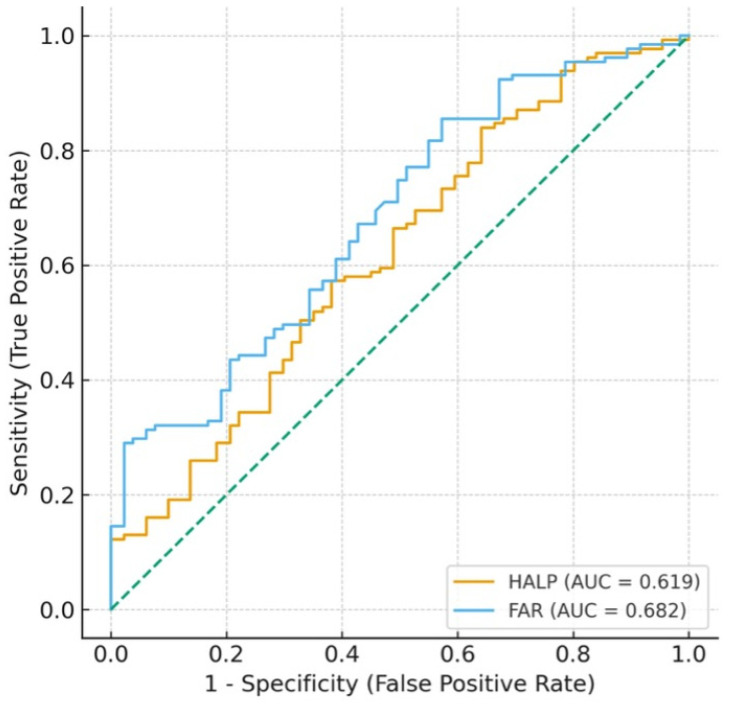
Receiver operating characteristic (ROC) curves of the HALP score and FAR for predicting preeclampsia. The FAR ratio demonstrated a higher discriminatory ability (AUC = 0.682) compared with the HALP score (AUC = 0.619). The diagonal dashed line represents the reference line of no discrimination. AUC: area under the curve.

Receiver operating characteristic curves (Figure 2) demonstrated superior discriminatory performance for FAR (AUC 0.682; 95% CI 0.618–0.751) compared with HALP (AUC 0.619; 95% CI 0.551–0.680).

Optimal cut-off values derived using Youden’s index were:

FAR ≥ 8.03 → Sensitivity 85.5%; specificity 42.7%.

HALP ≥ 28.37 → Sensitivity 84.0%; specificity 35.9%.

These findings indicate moderate diagnostic utility for both indices, with FAR consistently outperforming HALP (Table 3).

### 3.3. Logistic Regression Analysis

Because the primary objective of this study was to evaluate the independent predictive value of hematologic biomarkers (FAR and HALP), the multivariable logistic regression model included only these two indices rather than broader clinical covariates such as maternal age, BMI, or gestational age. This approach prevents over-adjustment and preserves the interpretability of biomarker-specific effects. Variance inflation factor measurements (VIF < 2) confirmed the absence of multicollinearity.

**Figure 3:**
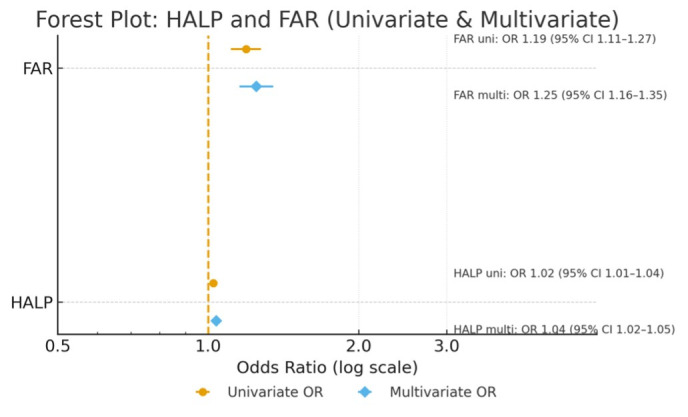
Forest plot showing the univariate and multivariate odds ratios (ORs) with 95% confidence intervals (CIs) for the association of the hemoglobin–albumin–lymphocyte–platelet (HALP) index and the fibrinogen-to-albumin ratio (FAR) with preeclampsia. Both markers were evaluated as continuous variables. FAR demonstrated a stronger and independent association with preeclampsia in multivariate analysis, whereas HALP showed a weaker but still significant effect. The vertical dashed line represents the null value (OR = 1), and ORs are presented on a logarithmic scale.

Univariate logistic regression demonstrated significant associations between both biomarkers and preeclampsia. Each one-unit increase in FAR was associated with a 20.6% increase in the odds of preeclampsia (OR 1.206; 95% CI: 1.124–1.295; *p* < 0.05), whereas the HALP score demonstrated a modest but significant association (OR 1.025; 95% CI: 1.010–1.039; *p* < 0.05). In the multivariable model including both indices, FAR remained a strong independent predictor (adjusted OR 1.263; 95% CI: 1.167–1.368; *p* < 0.05), while HALP retained a statistically significant but comparatively weaker association (adjusted OR 1.037; 95% CI: 1.020–1.054; *p* < 0.05). Model calibration was adequate according to the Hosmer–Lemeshow test (*p* > 0.05). Forest plot displays odds ratios derived from continuous-form logistic regression models (Figure 3).

### 3.4. Graphical Comparison of Biomarkers

**Figure 4:**
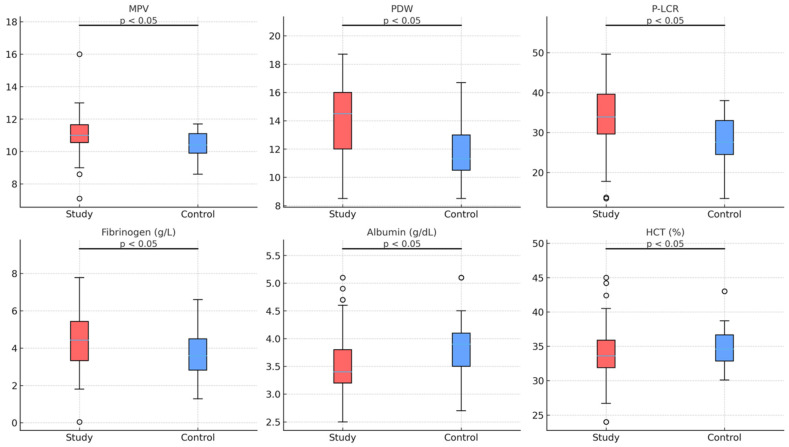
Boxplots comparing selected laboratory parameters—including MPV, PDW, P-LCR, fibrinogen, albumin, and hematocrit (HCT)—between the preeclampsia (study) and control groups. Study and control distributions are shown using color-coded boxplots (red for the study group and blue for the control group). Statistically significant differences between groups are indicated by the annotation “*p* < 0.05” positioned above each pair of boxplots. Boxes represent the interquartile range (IQR), horizontal lines within boxes denote median values, and whiskers indicate variability outside the upper and lower quartiles. Outliers are displayed as individual data points. Group differences were assessed using the Mann–Whitney U test. Significance levels are indicated as *p* < 0.05.

Boxplots illustrating significant biomarker differences between groups are shown in Figure 4. Women with preeclampsia exhibited higher platelet activation markers (MPV, PDW, and P-LCR), elevated fibrinogen and LDH levels, lower albumin concentrations, and reduced hematocrit values (all *p* < 0.05).

**Figure 5:**
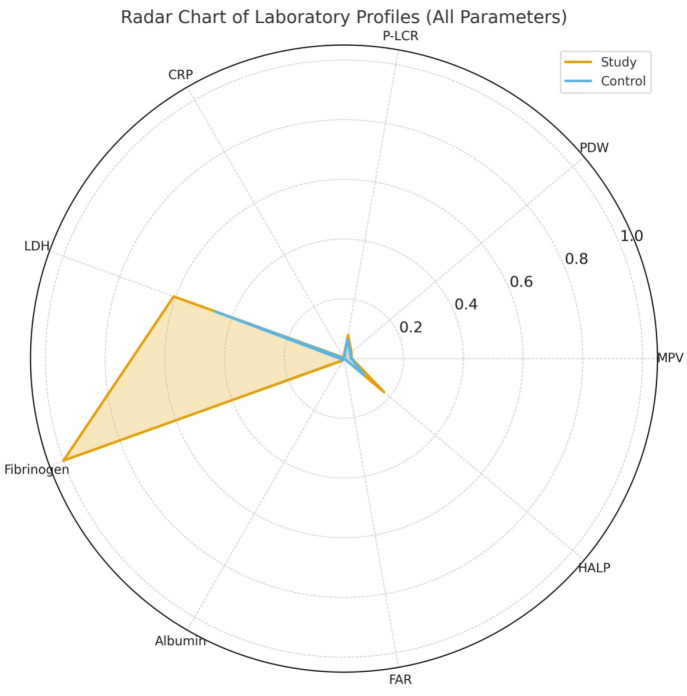
Radar chart comparing standardized laboratory profiles between the preeclampsia (study) and control groups. The study group demonstrated higher levels of inflammatory and coagulation-related biomarkers (LDH, fibrinogen, and FAR) and lower albumin levels. Platelet activation indices (MPV, PDW, and P-LCR) showed moderate group differences, while HALP exhibited minimal separation between groups. Values were normalized for visualization purposes.

A standardized radar chart (Figure 5) highlights the multidimensional divergence between groups, demonstrating disproportionately elevated inflammatory and coagulation biomarkers among preeclamptic pregnancies.

**Figure 6:**
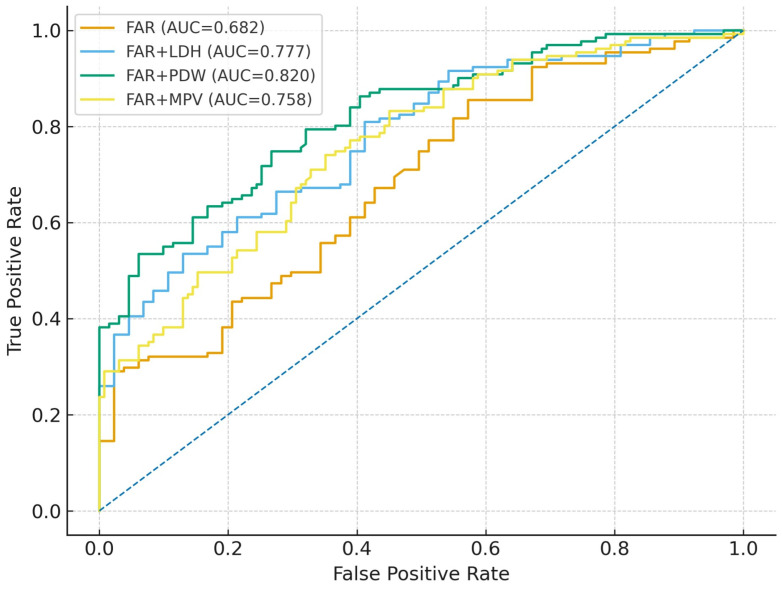
Receiver operating characteristic (ROC) curves comparing FAR alone with combined multivariable models incorporating LDH, PDW, or MPV. The FAR + PDW model demonstrated the highest discriminative ability (AUC = 0.820), followed by FAR + LDH (AUC = 0.777) and FAR + MPV (AUC = 0.758), outperforming FAR alone (AUC = 0.682). The improvement in AUC was statistically significant only for FAR + PDW compared with FAR alone (DeLong *p* = 0.030). The diagonal dashed line (blue) represents the reference line of no discrimination (AUC = 0.5).

In addition to single-marker ROC analyses, we constructed multivariable logistic regression models to evaluate whether combining FAR with selected biomarkers could improve discrimination (Figure 6). Compared with FAR alone (AUC 0.682; 95% CI 0.615–0.737), the FAR + LDH model yielded a higher AUC (0.777; 95% CI 0.716–0.830), with increased specificity (87.0%) at the expense of sensitivity (53.4%); however, this improvement did not reach statistical significance in DeLong’s test (*p* = 0.081) (Table 3). In contrast, combining FAR with PDW resulted in the best overall discrimination (AUC 0.820; 95% CI 0.770–0.862), with a more balanced sensitivity and specificity profile (74.8% and 73.3%, respectively). The AUC of the FAR + PDW model was significantly higher than that of FAR alone (DeLong *p* = 0.030). The FAR + MPV model produced an intermediate AUC (0.758; 95% CI 0.697–0.805), but its improvement over FAR alone was not statistically significant (*p* = 0.153).

## 4. Discussion

Preeclampsia remains a multifactorial disorder in which early placental dysfunction and the subsequent maternal systemic response interact to drive disease progression [9]. In this context, identifying accessible biomarkers that reflect these interconnected pathways is critical for improving risk stratification. The present study demonstrates that FAR and platelet activation markers are strongly associated with preeclampsia, whereas the HALP score offers limited standalone diagnostic utility. These findings support the growing emphasis on integrative biomarker approaches aimed at enhancing early prediction while maintaining feasibility in routine obstetric practice.

Our results confirm that women with preeclampsia exhibit significantly elevated fibrinogen, LDH, CRP, and D-dimer levels, accompanied by reduced serum albumin concentrations. These changes reflect a well-established biological cascade: systemic endothelial activation increases fibrinogen synthesis, capillary leakage reduces albumin, and placental hypoxia leads to cellular injury and the release of LDH and inflammatory cytokines. FAR, which mathematically and physiologically reflects the ratio of pro-inflammatory coagulation activity to plasma oncotic balance, therefore emerges as a biologically plausible marker of this dysregulated state. This is consistent with recent studies demonstrating that FAR is positively associated with endothelial dysfunction, oxidative stress, and adverse perinatal outcomes in hypertensive disorders of pregnancy [21,22,23]. The strong performance of the FAR in both ROC and multivariable models observed in this study is biologically plausible given the central role of both components in preeclampsia pathogenesis. FAR reflects the imbalance between procoagulant inflammatory activity and protective plasma homeostasis, providing an integrated representation of the pathophysiological processes underlying preeclampsia [24]. FAR therefore captures the imbalance between procoagulant inflammatory activity and protective plasma homeostasis, providing a more integrated reflection of disease severity than either marker alone.

From a diagnostic performance perspective, the observed AUC values warrant contextual interpretation. While the discriminative performance of FAR alone was moderate, it is important to interpret AUC values within the clinical context of preeclampsia, a heterogeneous syndrome influenced by multiple biological pathways. Single biomarkers rarely achieve high discrimination in isolation. Importantly, FAR demonstrated consistent directional associations across univariate, multivariable, and correlation analyses, supporting its biological relevance rather than standalone diagnostic precision. The marked improvement observed with the FAR + PDW model underscores the complementary value of combining coagulation–inflammation indices with platelet activation markers, a strategy increasingly advocated for in biomarker-based risk assessment.

Although several between-group differences in laboratory parameters were modest in absolute magnitude, the overall directional consistency across inflammatory, coagulation, and platelet activation markers supports a biologically coherent pattern characteristic of preeclampsia. Rather than isolated abnormalities, the concurrent elevation of fibrinogen, FAR, LDH, and platelet activation indices, alongside reduced albumin and platelet counts, suggests coordinated activation of interconnected pathophysiological pathways. These findings underscore the value of integrated biomarker assessment over reliance on single laboratory parameters when evaluating preeclampsia risk.

The HALP index, originally developed in oncologic populations, demonstrated only modest discriminatory capacity in our cohort. This limited performance likely reflects physiological hematologic adaptations of pregnancy—including hemodilution, gestational anemia, and platelet dynamics—which may distort the biological interpretation of HALP components. The paradoxical elevation of HALP appears predominantly driven by platelet reduction rather than proportional changes in hemoglobin or albumin, helping explain the inconsistent findings reported in the obstetric literature [9,18,25].

Although angiogenic biomarkers such as sFlt-1 and PlGF primarily reflect upstream placental dysfunction, composite hematologic indices may capture the downstream maternal systemic response. In this context, the relatively modest performance of the HALP score observed in our cohort may be attributable to the physiological hematologic adaptations of pregnancy, including plasma volume expansion and gestational changes in platelet dynamics, which can influence its individual components. Conversely, FAR directly integrates inflammatory and coagulation pathways that are more closely aligned with the maternal endothelial response characteristic of preeclampsia.

Rather than competing with established angiogenic markers, these hematologic biomarkers may provide complementary clinical insight, potentially enhancing risk stratification when used within a multimarker framework. This integrative perspective aligns with the growing recognition that complex disorders such as preeclampsia are unlikely to be predicted by a single biomarker.

Platelet indices provided additional mechanistic insight into the maternal vascular response. MPV, PDW, and P-LCR—markers of platelet activation and size variability—were significantly higher in preeclamptic pregnancies [26,27]. Among these markers, PDW demonstrated the greatest incremental value when combined with FAR, potentially capturing early microangiopathic alterations and platelet heterogeneity associated with hypertensive disorders of pregnancy.

The FAR + PDW model in our study achieved the highest AUC and was the only combination that significantly outperformed FAR alone, supporting the potential benefit of integrating coagulation–inflammation and platelet morphology metrics in future predictive models. Platelet activation represents a pivotal component of the microangiopathic process underlying preeclampsia [28]. Platelet distribution width (PDW) reflects anisocytosis and the presence of larger, more reactive platelets released from the bone marrow in response to peripheral consumption [29]. The observed incremental value of PDW when combined with FAR suggests that integrating platelet morphology with coagulation–inflammation indices enhances the detection of early microvascular dysfunction.

Importantly, these biomarkers should be considered complementary rather than standalone predictors within contemporary multifactorial screening strategies. By reflecting inflammatory and coagulation pathways not fully captured by traditional markers, FAR and PDW may provide incremental value; however, their integration into screening models requires prospective validation. The persistence of this association after adjustment for established maternal risk factors further supports the robustness of FAR as a biologically relevant biomarker.

Renal function markers also demonstrated clinically relevant trends. Although the absolute difference in serum creatinine was small, even modest elevations within the pregnancy-specific reference interval are clinically relevant and consistent with impaired renal perfusion in preeclampsia [30,31]. Given that normal pregnancy is characterized by enhanced glomerular filtration, slight creatinine rises may represent early glomerular injury or reduced renal blood flow, further reinforcing the systemic nature of preeclampsia-associated end-organ dysfunction [32].

The correlation analyses provide additional coherence to the biological framework of our findings. Strong positive correlations among fibrinogen, FAR, and D-dimer underscore the interplay between inflammatory and coagulation pathways, while robust intercorrelations among platelet indices reflect intensified platelet activation and consumption. In contrast, HALP demonstrated weak associations with all biomarkers, reinforcing its limited mechanistic alignment with preeclampsia pathophysiology. This divergence highlights the importance of selecting biomarkers grounded in the disease’s dominant biological pathways rather than relying on broader indices derived from non-pregnant populations.

From a clinical perspective, FAR and platelet activation markers are derived from routinely available laboratory parameters and may offer practical insights into the biological processes underlying preeclampsia. However, these biomarkers should be viewed as complementary rather than standalone predictors within contemporary multifactorial screening strategies.

Although the FAR + PDW model demonstrated improved discriminative performance, the findings should be interpreted within the context of several limitations. First, the retrospective single-center design may limit generalizability. Second, perinatal outcome data were not systematically available due to a transition in the institutional electronic medical record system during the study period, restricting access to complete neonatal datasets. However, the primary objective of this study was to evaluate the predictive performance of hematologic biomarkers rather than neonatal outcomes.

Additionally, established angiogenic biomarkers such as sFlt-1 and PlGF were not routinely measured in our institution during the study period, precluding direct comparative analyses. Additionally, residual confounding inherent to retrospective observational designs cannot be entirely excluded. Nevertheless, our findings suggest that hematologic biomarkers may offer complementary insight into the downstream maternal response to placental dysfunction.

Future large-scale prospective studies integrating angiogenic and inflammatory biomarkers may help determine whether multimarker models further improve predictive accuracy. Until such evidence emerges, these markers should be considered investigational rather than ready for routine clinical implementation.

This study demonstrates that FAR—particularly when combined with PDW—may serve as a promising biomarker reflecting the inflammatory and coagulation disturbances underlying preeclampsia. Although HALP differed statistically between groups, its weak correlations and limited mechanistic specificity suggest a lower predictive contribution compared with coagulation–inflammation indices. Importantly, these biomarkers should not be interpreted as standalone screening tools but rather as complementary parameters that may enhance existing risk assessment frameworks. While the combined FAR + PDW model showed improved discriminatory performance, these findings should be considered preliminary. Prospective, multicenter studies with external validation are required to confirm their clinical relevance before integration into routine obstetric practice can be considered.

## Figures and Tables

**Table 1:** Baseline demographic characteristics of the groups.

Variable	Control Group (*n* = 131)	Preeclampsia Group (*n* = 131)	*p*-Value
Maternal age (years)	28.76 ± 4.81	28.55 ± 4.46	0.713
BMI (kg/m^2^)	29.97 ± 3.45	29.93 ± 2.65	0.916
Gravidity	1.81 ± 0.81	1.45 ± 0.77	<0.05
Parity	0.77 ± 0.80	0.41 ± 0.73	<0.05
Abortus (*n*)			
0	126 (96.2%)	126 (96.2%)	1.000
1	5 (3.8%)	5 (3.8%)	1.000
≥2	0	0	-
SBP (mmHg)	113.17 ± 8.42	149.97 ± 13.23	<0.05
DBP (mmHg)	72.34 ± 5.03	94.50 ± 6.53	<0.05
Gestational age at delivery (weeks)	37.90 ± 1.57	37.27 ± 1.04	<0.05
Cesarean delivery, *n* (%)	92 (70.2%)	123 (93.2%)	<0.05
Vaginal delivery, *n* (%)	39 (29.8%)	8 (6.8%)	<0.05
Smoking, *n* (%)	12 (9.1%)	21 (15.9%)	<0.05
Alcohol, *n* (%)	2 (1.5%)	5 (3.8%)	<0.05
Birthweight (g)	2915.30 ± 369.53	2852.85 ± 359.34	0.1667
Birth length (cm)	49.10 ± 1.78	49.05 ± 2.53	0.8655

Comparison of maternal demographic, obstetric, delivery, and neonatal characteristics between the control and study groups. Continuous variables are presented as mean ± standard deviation and compared using Welch’s *t*-test. Categorical variables are expressed as number (percentage) and compared using Fisher’s exact test.

**Table 2:** Laboratory findings of study and control groups.

Parameter	Preeclampsia Group (Mean ± SD)	Control Group (Mean ± SD)	*p*-Value
Hgb (g/L)	113.95 ± 12.01	116.69 ± 9.44	<0.05
Fibrinogen (g/L)	4.26 ± 1.44	3.62 ± 1.07	<0.05
LDH (U/L)	258.49 ± 99.60	195.73 ± 48.59	<0.05
Platelets (×10^3^/µL)	187.03 ± 58.94	226.39 ± 42.41	<0.05
Creatinine (mg/dL)	0.57 ± 0.12	0.50 ± 0.08	<0.05
D-dimer (mg/L)	1.09 ± 0.84	0.70 ± 0.57	<0.05
CRP (mg/L)	1.51 ± 3.49	0.13 ± 0.21	<0.05
Hematocrit (%)	33.94 ± 3.52	34.72 ± 2.76	<0.05
ALT (U/L)	15.66 ± 12.36	18.13 ± 13.20	0.1382
AST (U/L)	22.51 ± 11.53	24.04 ± 10.33	0.0611
P-LCR (%)	34.15 ± 7.36	28.15 ± 6.17	<0.05
MPV (fL)	11.10 ± 1.06	10.46 ± 0.76	<0.05
PDW (fL)	13.98 ± 2.33	11.84 ± 1.67	<0.05
Albumin (g/dL)	3.51 ± 0.48	3.85 ± 0.45	<0.05
Lymphocytes (×10^3^/µL)	1.99 ± 0.61	1.80 ± 0.57	0.4189
HALP score	47.23 ± 25.56	37.73 ± 16.51	<0.05
FAR	12.44 ± 4.43	9.63 ± 3.27	<0.05

Comparison of laboratory parameters between the study and control groups. Data are presented as mean ± standard deviation. A *p*-value < 0.05 was considered statistically significant.

**Table 3:** Diagnostic performance of FAR alone and in combination with LDH, PDW, and MPV for predicting preeclampsia.

Model	AUC (95% CI)	Sensitivity (%)	Specificity (%)	Cut-Off (Youden)	DeLong *p* vs. FAR
FAR	0.682 (0.615–0.737)	85.5	42.7	≥8.03	–
FAR + LDH	0.777 (0.716–0.830)	53.4	87.0	≥0.412 *	0.081
FAR + PDW	0.820 (0.770–0.862)	74.8	73.3	≥0.394 *	0.030
FAR + MPV	0.758 (0.697–0.805)	74.0	64.9	≥0.381 *	0.153

Area under the ROC curve (AUC), 95% confidence intervals (CIs), optimal cut-off values (based on Youden’s index), sensitivity, specificity, and DeLong *p*-values comparing each combined model with FAR alone are presented. The FAR + PDW model demonstrated the greatest improvement in discrimination, with a significantly higher AUC compared with FAR alone (DeLong *p* = 0.030). Cut-off values marked with (*) represent the optimal predicted probability thresholds obtained from multivariable logistic regression models.

## Data Availability

Dataset available on request from the authors.
